# The Swedish childhood cancer quality registry for risk-based long-term follow-up after childhood cancer: structure, content, and development over time

**DOI:** 10.1186/s13690-026-02136-2

**Published:** 2026-11-09

**Authors:** Cecilia Petersen, Päivi Lähteenmäki, Madeleine Helmersson, Lars Hjorth, Marianne Jarfelt, Emma Nordh, Per Nyman, Margaretha Stenmarker, Aron Onerup, Anna S. Holmqvist

**Affiliations:** 1 Childhood Cancer Research Unit, Department of Women’s and Children’s Health, Karolinska Institutet, Stockholm, Sweden; 2 Regional Cancer Center West, Western Sweden Health Care Region, Gothenburg, Sweden; 3 Department of Clinical Sciences, Faculty of Medicine, Lund University, Lund, Sweden; 4 Department of Oncology, Institute of Clinical Sciences, Sahlgrenska Academy, University of Gothenburg, Gothenburg, Sweden; 5 Department of Biomedical and Clinical Sciences, Linköping University, Linköping, Sweden; 6 Department of Pediatrics, Institute of Clinical Sciences, Sahlgrenska Academy, University of Gothenburg, Gothenburg, Sweden; 7 Department of Pediatric Oncology, Sahlgrenska University Hospital, Gothenburg, Sweden; 8 Childhood Cancer Center, Skåne University Hospital, Lund, Sweden; 9 Pediatric Oncology Unit, Karolinska University Hospital, Stockholm, Sweden; 10 Crown Princess Victoria Children´s Hospital, Linköping University Hospital, Linköping, Sweden

**Keywords:** Childhood cancer, Quality registry, Fertility, Cardiotoxicity, Survivor

## Abstract

**Background:**

Survival after childhood cancer has increased dramatically during the last 50–60 years. Today, approximately 86% of childhood cancer patients in Sweden survive more than 5 years, and most of them reach adulthood. The intense multimodal cancer treatment is unfortunately associated with a risk of late complications during childhood or later in adult life. A registry for monitoring late complications is an important tool to support structured long-term follow-up. In Sweden this is available through the SALUB database, a part of the Swedish childhood cancer quality registry. The aim of this study is to describe the SALUB database and assess its coverage, its capacity to identify treatment-defined risk groups for late complications, and the completeness of long-term follow-up data.

**Methods:**

A cohort of patients who had reached at least 19 years of age, were born before 01/07/2006, and had received or were assumed to have received systemic cancer treatment or radiotherapy was identified in the Swedish childhood cancer quality registry. Database coverage was assessed by individual-level linkage, using personal identity numbers, between the SALUB database and the diagnosis-based databases. Subpopulations were assessed to explore changes over time, identify treatment-defined risk group, and assess the completeness of follow-up data.

**Results:**

The coverage was 73% (3,780/5,171) in the overall cohort and 94% (803/853) in patients born between 2002 and 2006. The number of individuals at risk for cardiotoxicity and gonadal toxicity were easily identified. A total of 1,809 patients had at least one follow-up record meeting the inclusion criteria. Data completeness varied markedly across organ systems.

**Conclusions:**

The SALUB database has high coverage among younger survivors and enables identification of individuals according to treatment-defined risks of late complications. However, lower coverage in older survivors and incomplete follow-up data currently limit some of its potential applications. Continued development of the database will strengthen its role in risk-based survivorship care, health care planning, and research.

**Supplementary Information:**

The online version contains supplementary material available at https://doi.org/10.1186/s13690-026-02136-2.

**Table:** 

Text box 1. Contributions to the literature	
• Provides the first detailed description of the Swedish national population-based registry for long-term follow-up of childhood cancer survivors. • Illustrates how a national database for long-term follow-up can be integrated into clinical survivorship care after childhood cancer. • Highlights the potential of registry-based risk stratification to support health care quality monitoring, resource planning, and the identification of research priorities. • Provides an example on how continuity of information can be maintained during transition from pediatric to adult long-term follow-up.	

## Background

Improvements in childhood cancer treatment have resulted in a growing population of long-term survivors. In Sweden, approximately 86% of childhood cancer patients survive more than 5 years, and most of them reach adulthood [1]. The success story of intense multimodal cancer treatment is unfortunately associated with a risk of late complications [2–10]. These late complications may arise already during childhood and adolescence as well as later in life. Structured long-term follow-up is therefore an important component of survivorship care, requiring access to detailed information on previous treatment exposures and subsequent health outcomes.

This paper is a registry description of the structure and content of the Swedish national database for long-term follow-up after childhood cancer, the SALUB database. We assess registry coverage across different birth cohorts by comparing the data in the SALUB database with data from the diagnosis-based databases in the Swedish childhood cancer quality registry. We also describe the completeness of follow-up data at the individual level, based on each patient´s latest available follow-up record. Finally, we aim to illustrate how treatment exposure data in the database can be used to identify the number of individuals potentially at risk of late effects based on exposure to specific cumulative doses of chemotherapeutic agents or radiation doses. By describing the content and limitations of the registry, this paper aims to facilitate the appropriate interpretation of registry data in future research and to illustrate the registry’s potential utility for planning research projects and follow-up services.

### Long-term follow-up guidelines

Guidelines for long-term follow-up (LTFU) have been established worldwide and harmonized through the collaborative work by the International Guideline Harmonization Group for Late Effects of Childhood Cancer (IGHG) [11, 12]. In Sweden, a national care program for long-term follow-up after childhood cancer was first published in 2016 [13]. These national guidelines describe the modality and frequency of recommended follow-up investigations for specified organs at risk, largely based on the IGHG guidelines or, when IGHG publications are lacking, other evidence- or consensus-based recommendations. The national care program aims to adapt the international recommendations to the Swedish health care system and to other national health care guidelines.

Despite comprehensive guidelines, evidence on the benefits of many surveillance investigations of organ-specific toxicity remains limited. Systematic registration of detailed treatment exposures and surveillance outcomes is needed to evaluate the yield of recommended investigations, characterize the landscape of late complications, and inform future follow-up guidelines. Such data also provide the basis for research within the field of late complications.

### Structure of long-term follow-up in Sweden

**Fig. 1:**
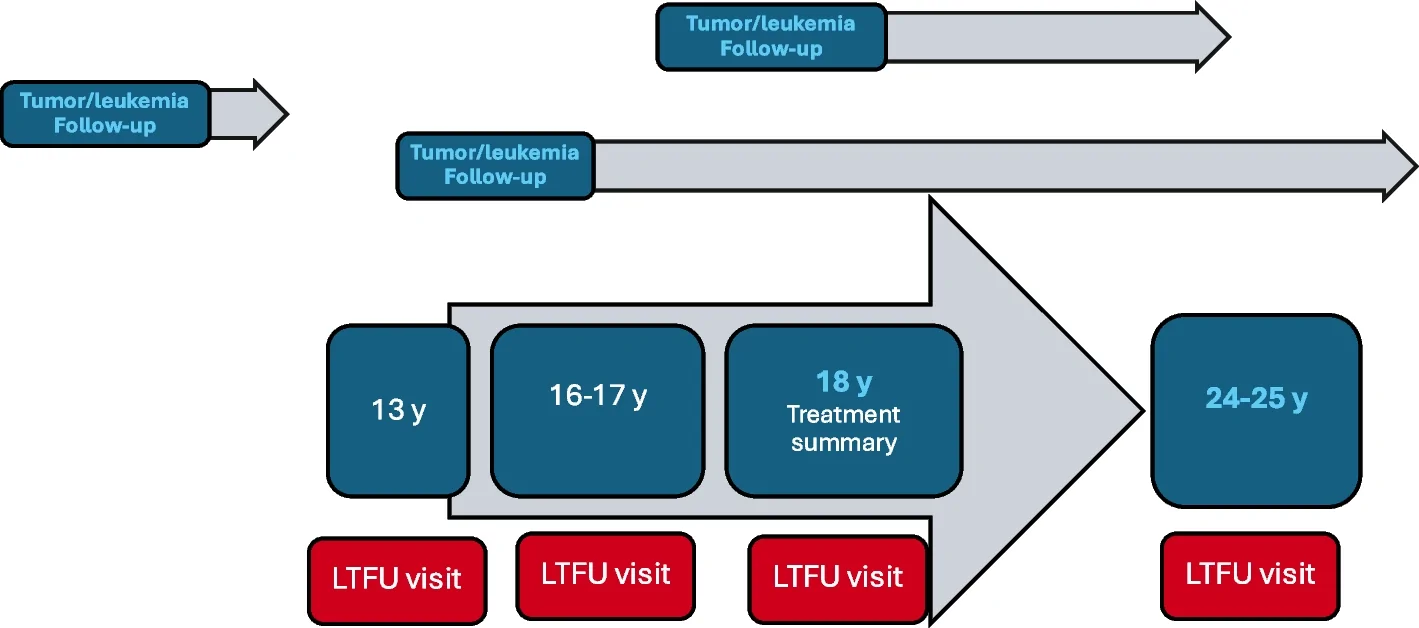
Structure of long-term follow-up in Sweden. Schematic illustration of long-term follow-up (LTFU) visits across different ages (y). LTFU visits may take place in parallel with disease-specific oncological follow-up. Additional LTFU visits are offered when clinically indicated

The national care program specifies the organizational structure for long-term follow-up clinics during adolescence and adulthood and is revised every second to third year. During adolescence, 2–3 LTFU visits, starting at age 13, should be offered after the end of treatment to all patients having received systemic cancer treatment or radiotherapy. Among several objectives, these LTFU visits play an important role in the transition to adult care. Depending on the type of diagnosis and age at the end of treatment, the LTFU visits may take place in parallel to disease-specific oncological follow-up (Fig. 1). At age 18, patients are referred to the LTFU clinic for adults after childhood cancer (LTFU-ACC), where they are offered a first visit at age 23–25, or earlier if needed. Patients treated with surgery only may also be referred when considered appropriate. The LTFU-ACC continues to offer the patients regular visits if there is a need for specific check-ups or screening according to the national care program, if no other caregiver is considered more appropriate. These routines for further visits at the LTFU-ACC may vary between the different health care regions in the country. All patients, even those not needing regular follow-up investigations for specific organ toxicity, are thus offered at least one visit at the LTFU-ACC.

The LTFU program for teenagers and adults was fully implemented across all six childhood cancer centers and LTFU-ACC in 2020. Consequently, some adult childhood cancer survivors have not yet attended an LTFU clinic. Survivors who become aware of the adult LTFU clinics are encouraged to contact them regardless of their age. Thus, some patients are in their 50 s or 60 s at their first visit.

### The SALUB database

Around 2010, the Swedish working group for long-term follow-up after childhood cancer (SALUB) started to develop variables for detailed treatment data registration, the SALUB database, a part of the Swedish childhood cancer quality registry [1, 14]. The registry is composed of additional databases, related to the different diagnosis groups of leukemia, lymphoma, solid tumors and CNS tumors, respectively, as well as separate databases covering radiotherapy and rehabilitation. The first patient in the SALUB database was registered in 2012. At that time, the primary aims of the database were to generate survivorship passports from the database, treatment summaries with individualized follow-up recommendations for adolescents transitioning to adult care, and to retain detailed treatment data for future use. Survivorship passports had already been provided to patients for several years, but only in paper format and without a system for storing the underlying detailed treatment data.

In the SALUB database, all chemotherapeutic agents, including cumulative doses (most commonly per m^2^), other systemic anticancer treatments, radiotherapy exposures to specific organs, dates of surgical procedures, and the start and end dates for systemic treatment, radiotherapy and stem cell transplantation, are recorded. The number of variables included in the database has increased substantially since its establishment in 2012.

In 2022, the database was transferred to the common registry platform for Swedish cancer quality registries, enabling improved data quality monitoring. Since then, the database includes reliable information on the number of survivorship passports generated, and whether they have been provided to patients at a pediatric and/or adult LTFU clinic.

In 2022, an improved follow-up report form was added as part of routine clinical practice. Data are manually recorded based on information in patient´s medical records from regular visits to the LTFU clinic. In addition to providing detailed information on the health status of the patient, completion of the form can serve as an indicator that an LTFU visit has taken place, as the form is intended to be completed at each such visit. This allows individual clinics to monitor their activity over time and compare it with national practice. The follow-up form further contains information on what follow-up investigations have been performed, an evaluation of whether the national care program has been adhered to or not (totally, partially or not at all) and if there is a need for revised recommendations for the patient. From 2022 and onwards, the follow-up recommendations are automatically generated, while there is still a possibility to bypass the automatically generated texts to create more personalized recommendations. The automatic texts are harmonized with the national care program and thus need to be revised when a new version of the latter is published.

In addition to supporting clinical practice at LTFU clinics, a major long-term goal of the database is to enable evaluation of current follow-up recommendations and to provide data that can inform future revisions of the national care program. Another objective is to continuously measure the adherence to the national care program recommendations as a health care quality indicator.

The SALUB database may also provide an important resource for research on associations between treatment exposures and late complications. In Sweden, the availability of national registries with high coverage and validity [15–18], together with the use of unique personal identification numbers, provides opportunities for linkage of SALUB data with other registry-based data sources.

#### General aspects of the SALUB database

The goal is for all childhood cancer patients treated with systemic anticancer therapy, stem cell transplantation and/or radiotherapy to have detailed treatment data registered the SALUB database by 18 years of age. The database is continuously being developed, and the follow-up form has been revised several times since 2022. Most recently, variables covering additional organ systems at risk were added to the follow-up form. Consequently, these newly introduced variables are incomplete for patients who have not attended an LTFU visit since their introduction. As additional follow-up visits are recorded, the completeness of these variables is expected to improve. Understanding how and when data are entered into the SALUB database is important for interpreting its coverage and completeness.

#### When is patient data registered in the SALUB database?

Treatment exposure data may be entered into the SALUB database by either the pediatric oncology unit or the LTFU-ACC. Pediatric oncology units aim to register all patients who have received systemic anticancer treatment and/or radiotherapy, including detailed information on treatment exposures. Patients treated with surgery only are not systematically registered in the SALUB database since information on these patients is available in the diagnosis-based databases within the Swedish childhood cancer quality registry. If an eligible patient is not already registered, the LTFU-ACC enters the patient data before the first visit.

In 2020, a substantial number of patients from a regional population-based database in southern Sweden were added to the SALUB database, increasing in the number of patients treated in the 1970 s, 1980 s and 1990 s. The regional database included all five-year survivors diagnosed with childhood cancer from 1970 onward, regardless of treatment modality. Consequently, the transfer also introduced a substantial number of patients treated with surgery only into the SALUB database.

More recently, some pediatric oncology units have started registering patients in the SALUB database directly after the end of treatment, as detailed information on treatment exposures guides the follow-up plan also during childhood and adolescence. Consequently, patients may be registered in the SALUB database several years before transition to adult LTFU care.

**Fig. 2:**
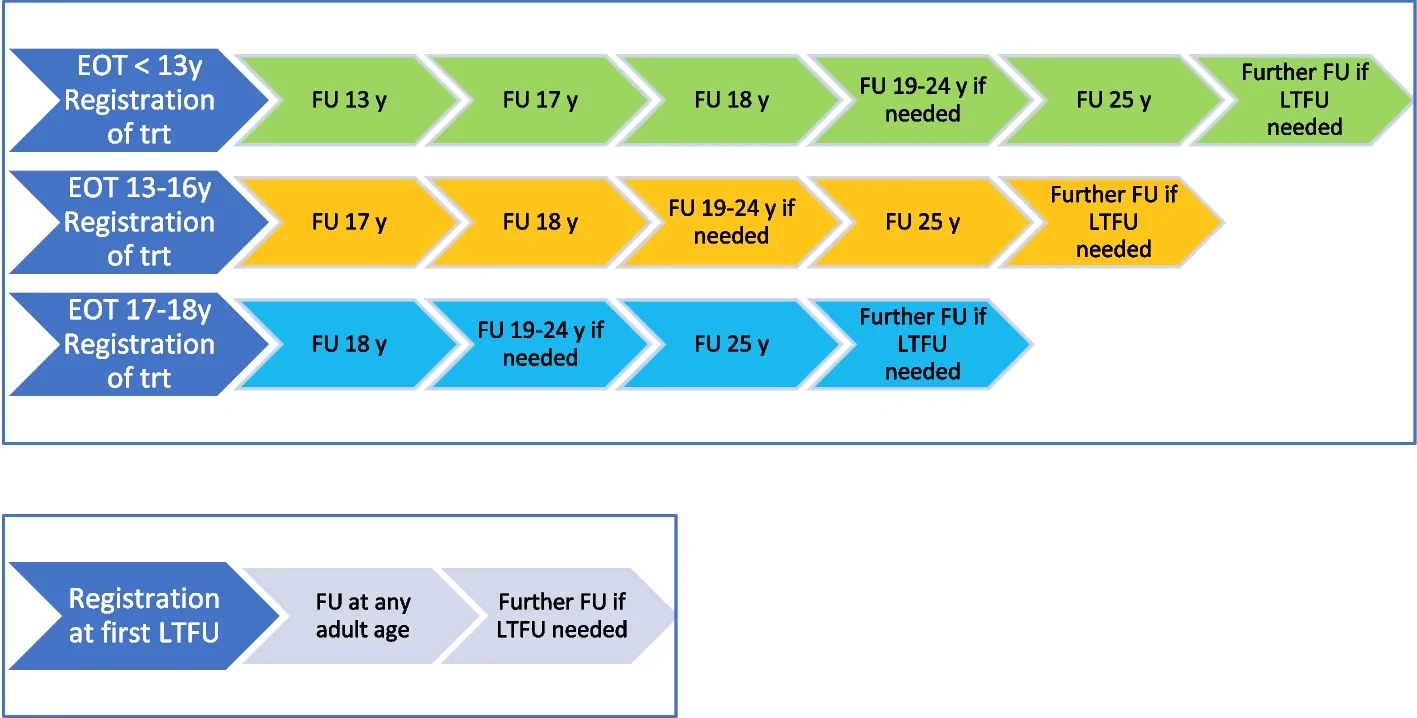
Timing of treatment and follow-up registration in the SALUB database according to year of birth. Upper panel: Expected registration for individuals born in 2004 or later. Treatment (trt) data are registered at the end of treatment (EOT) or at the first long-term follow-up (LTFU) visit, followed by follow-up (FU) registrations at different ages (y) depending on age at the EOT. Lower panel: Possible registration pattern for individuals born before 2004, for whom treatment and follow-up data may first be entered at an adult LTFU visit

The follow-up forms are intended to be completed at all LTFU visits during both adolescence and adulthood. The number of follow-up forms available for an individual patient therefore depends on the number of LTFU visits attended since the introduction of the form in 2022. The long-term aim is for each living patient to have at least three to four follow-ups forms completed by age 25 (Fig. 2).

### Questions addressed in the present study.

Given the timing of its establishment and the organization of long-term follow-up, the SALUB database is expected to have uneven coverage across age groups, with lower coverage among older survivors. Understanding the current strengths and limitations of the database is therefore essential for appropriate interpretation of its data.

Key questions to be considered:

1. What is the coverage of the SALUB database in relation to the diagnosis-based databases within the Swedish childhood cancer quality registry?
  - In total
  - In different age groups
  - In different diagnosis-based groups
2. How many patients in the SALUB database are not registered in the diagnosis-based databases?
3. Can the SALUB database be used to define the number of patients at risk for specific late complications?
4. Can any general conclusions be drawn from the follow-up data at present?

## Methods

To assess the coverage of the SALUB database, we defined an eligible study population using the complete datasets from the diagnosis-based databases and the SALUB database within the Swedish childhood cancer quality registry. As the SALUB database itself does not apply strict inclusion and exclusion criteria, predefined selection criteria were used to identify individuals expected to be eligible for the LTFU program and thus for registration in the database. The data extraction date was set to 30/06/2025. Selection criterion A was designed to identify the overall population expected to be eligible for registration in the SALUB database: individuals who were alive at age 19, were born before July 1 st, 2006, and had received or were assumed to have received systemic anticancer treatment and/or radiotherapy (Table 1). Selection criterion B applied the same treatment criteria but was restricted to individuals born between January 1, 2002, and June 30, 2006. This younger cohort was selected since the oldest individuals in this subcohort reached age 18 in 2020, when the national LTFU program was considered fully implemented. If information on treatment was missing, additional criteria with a list of ICCC-3 diagnoses considered to be associated with systemic treatment and/or radiotherapy were used as a proxy. These diagnoses were selected because, according to standard treatment practices, patients with these diagnoses rarely achieve successful treatment without systemic anticancer therapy or radiotherapy. Individuals were matched across the SALUB database and the diagnosis-based databases using their Swedish personal identity numbers. For individuals with multiple diagnoses in the diagnosis-based databases meeting the selection criteria for inclusion in the SALUB database, the earliest diagnosis was selected based on the date of diagnosis. Selection criteria A and B (Table 1) were applied to answer question no 1 above. To assess the accuracy of the coverage selection criteria, the age criteria from Selections A and B were also applied directly to the SALUB database. Treatment information recorded in the SALUB database was then used to identify individuals who were not captured by the selection algorithm applied to the diagnosis-based databases, as well as individuals who were captured despite having no recorded systemic anticancer treatment or radiotherapy. The Selection A age criteria were also used to identify individuals registered exclusively in the SALUB database and not in the diagnosis-based databases.

**Table 1:** Selection criteria for calculations of coverage

Selection A criterion	Selection B criterion
Alive at age 19	Alive at age 19
Date of birth ≤ June 30, 2006	Date of birth between 01/01/2002 and 30/06/2006
at least one of the following: • Registered chemotherapy, radiotherapy, or other systemic anticancer treatment OR • Selected ICCC-3 diagnosis used as a proxy for treatment exposure when treatment information was unavailable: I-II (leukemia and lymphoma) III a and III c (ependymoma, choroid plexus carcinoma and embryonal tumors in the central nervous system) ICCC3 VIa (nephroblastoma) ICCC3 VIIa (hepatoblastoma) ICCC3 VIIIa och VIIIc (osteosarcoma and Ewing sarcoma) ICCC3 IXa (rhabdomyosarcoma) ICCC3 Xa (germ cell tumors in the central nervous system) ICCC3 XIc (nasopharyngeal carcinoma)	at least one of the following: • Registered chemotherapy, radiotherapy, or other systemic anticancer treatment OR • Selected ICCC-3 diagnosis used as a proxy for treatment exposure when treatment information was unavailable: I-II (leukemia and lymphoma) III a and III c (ependymoma, choroid plexus carcinoma and embryonal tumors in the central nervous system) ICCC3 VIa (nephroblastoma) ICCC3 VIIa (hepatoblastoma) ICCC3 VIIIa och VIIIc (osteosarcoma and Ewing sarcoma) ICCC3 IXa (rhabdomyosarcoma) ICCC3 Xa (germ cell tumors in the central nervous system) ICCC3 XIc (nasopharyngeal carcinoma)

To assess whether the SALUB database could be used to identify the number of individuals at risk of specific late effects, predefined treatment exposure criteria for gonadal toxicity and cardiotoxicity were applied. International guidelines identify a cyclophosphamide equivalent dose (CED) of ≥ 6,000 mg/m^2^ and ovarian radiotherapy exposure > 10 Gy as markers of increased risk of impaired fertility in female survivors [19, 20]. For male survivors, a CED ≥ 4,000 mg/m^2^ or radiotherapy involving the testes has been associated with a high risk of treatment-related infertility [21]. Since organ-specific radiotherapy doses were not consistently available in the SALUB database, and both autologous and allogeneic stem cell transplantation involve either high-dose alkylating agents or total body irradiation, the risk-group algorithm used the sex-specific CED thresholds, stem cell transplantation, or radiotherapy to predefined anatomical targets as proxies for high-risk exposure. The equivalent doses were calculated using the following conversion factors: cyclophosphamide 1; ifosfamide 0.244; procarbazine 0.857; chlorambucil 14.286; carmustine 15; lomustine 16; melphalan 40; thiotepa 50; mustine 100; busulfan 8,823 [19]. The treatment exposures considered to be associated with a high-risk of cardiotoxicity are cumulative doxorubicin equivalent doses (DED) of ≥ 250 mg/m^2^ and/or radiotherapy exposure to the heart [11]. The treatments considered to be associated with a moderate risk are DED 100–249 mg/m^2^ or very low radiotherapy doses. Since the information on exact radiotherapy exposures to risk organs is far from complete for older age groups in the SALUB database, the selection criteria concerning radiotherapy was based on target organ and not on specific exposure dose. Craniospinal irradiation with photons and radiotherapy targets in thorax, were used as proxies for high-risk radiotherapy exposure. The equivalent doses were calculated using the following conversion factors: doxorubicin 1; daunorubicin 0.6; epirubicin 0.8; idarubicin 5; mitoxantrone 10.5 [22].

The specified selection criteria used to identify the number of individuals at risk for gonadal toxicity and cardiotoxicity are listed in Table 2.

**Table 2:** Selection criteria to identify individuals with high risk of gonadal toxicity and moderate to high risk of cardiotoxicity

High risk of gonadal toxicity	
Females	Males
Female gender	Male gender
Alive at age 19	Alive at age 19
At least one of the following: • Cyclophosphamide equivalent dose ≥ 6000 mg/m^2^ • Stem cell transplant • Radiotherapy to at least one of following targets: o Total body o Abdomen o Pelvis o Ovaries o Vagina	At least one of the following: • Cyclophosphamide equivalent dose ≥ 4000 mg/m^2^ • Stem cell transplant • Radiotherapy to at least one of following targets: o Total body o Testis
Risk of cardiotoxicity	
High risk	Moderate risk
Alive at age 19	Alive at age 19
At least one of the following: • Doxorubicin equivalent dose ≥ 250 mg/m^2^ • Radiotherapy to at least one of the following radiotherapy targets: o Total body o Thorax or Thoracic organs o Craniospinal irradiation with photons	• Doxorubicin equivalent dose > 100 < 250 mg/m^2^ • None of the following radiotherapy targets: o Total body o Thorax or Thoracic organs o Craniospinal irradiation with photons

For the analysis of follow-up data, we included all patients in the SALUB database who met the Selection criterion A and had at least one follow-up record at age 19 or older. For each individual, the most recent eligible follow-up record was analyzed. A follow-up record was considered eligible if the cardiac disease variable had been completed as “yes”, “no”, or “unknown”. This variable was selected because it has been included in the follow-up form since its introduction in 2022. It was therefore used as an indicator of a completed follow-up record rather than as a clinical inclusion criterion.

## Results

As of 30 June 2025, 13 years after the first registered patient, the SALUB database contained 7780 patients aged 0 − 75 years. Among patients alive, the median age was 26 years (IQR 19–36 years).

### Coverage analysis (questions 1 a-c)

**Fig. 3:**
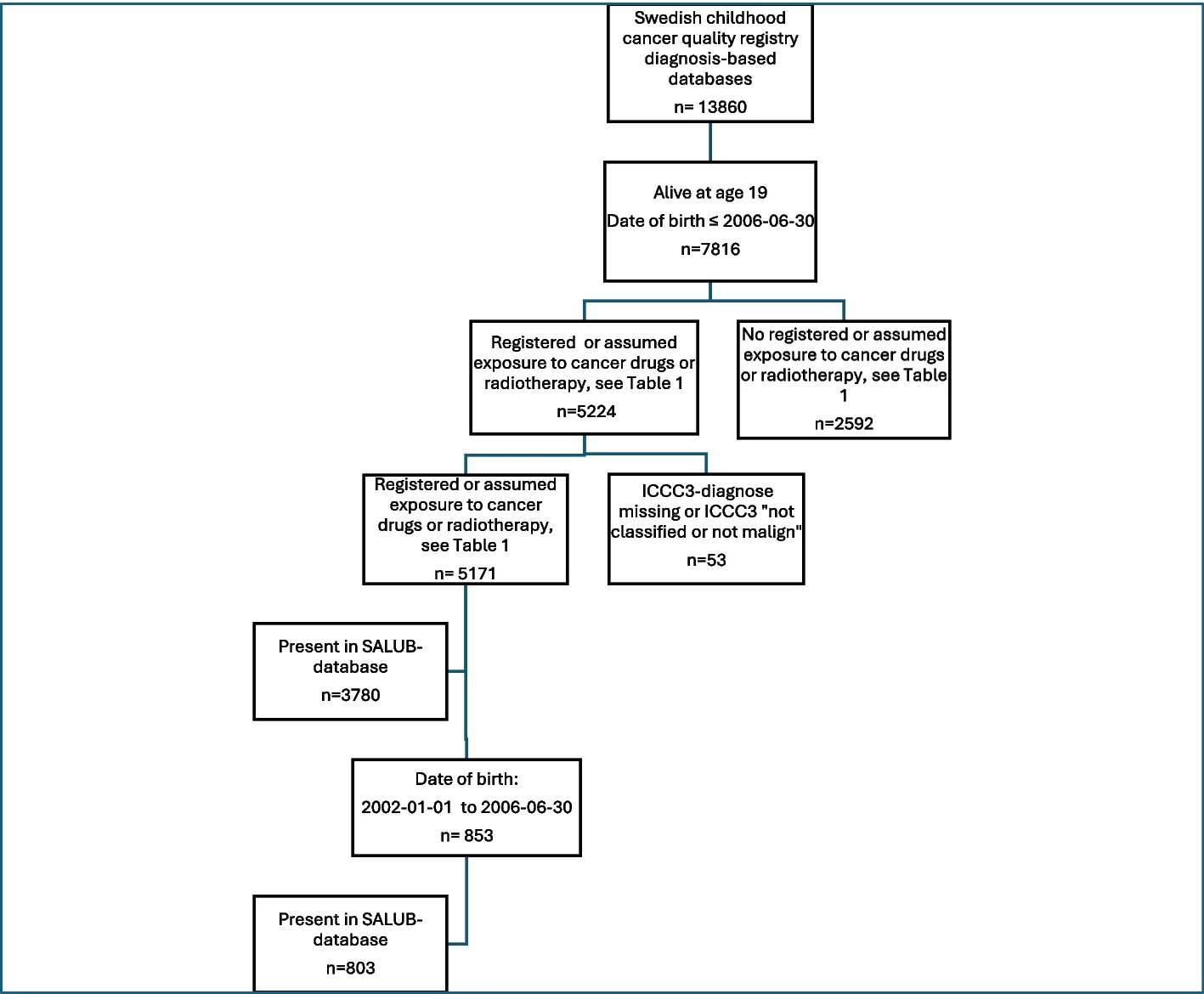
Selection of the study population. Flow diagram showing the number of individuals identified in the diagnosis-based databases of the Swedish childhood cancer quality registry using Selection criteria A and B (Table 1), and the number of these individuals also registered in the SALUB database

Using Selection criterion A, 5,171 individuals in the diagnosis-based modules were identified as eligible for registration in SALUB. Of these, 3,780 were present in the SALUB database (Fig. 3), corresponding to an overall coverage of 73.1%.

**Fig. 4:**
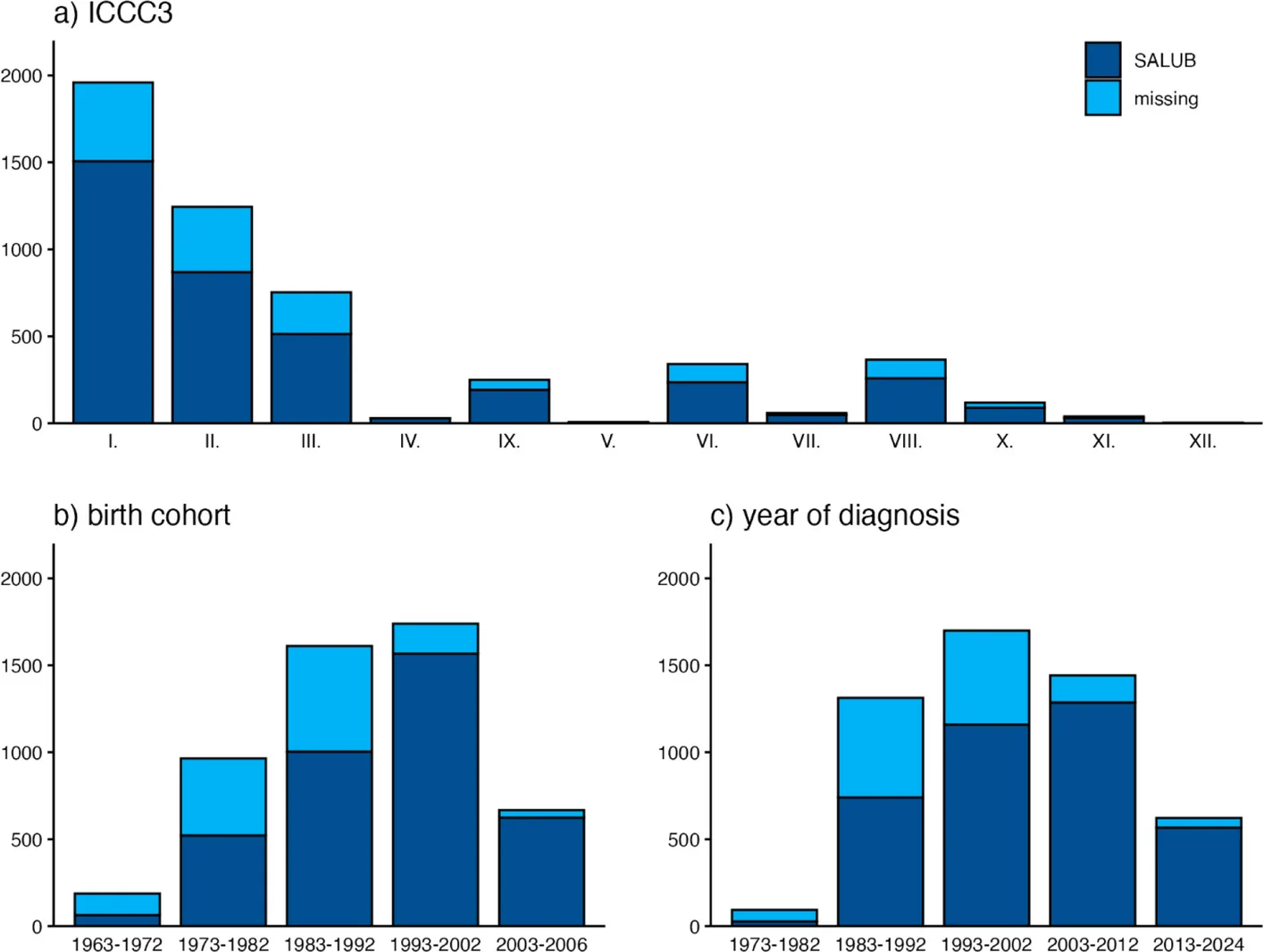
Coverage of the SALUB database. Coverage according to ICCC-3 diagnostic group (**a**), birth cohorts (**b**), and year of diagnosis (**c**) when Selection A criterion (Table 1) was applied. Light blue color indicates the number of missing individuals. The classification according to ICCC-3–2017 is provided in the foot notes. Foot notes: I=Leukemias, myeloproliferative and myelodysplastic diseases, II = Lymphomas and reticuloendothelial neoplasms, III = CNS and miscellaneous intracranial and intraspinal neoplasms, IV = Neuroblastoma and other peripheral nervous cell tumors, IX = Soft tissue and other extraosseous sarcomas, V = Retinoblastoma, VI = Renal tumors, VII = Hepatic tumors, VIII = Malignant bone tumors. X = Germ cell tumors, trophoblastic tumors and neoplasms of gonads, XI = Other malignant epithelial neoplasms and malignant melanomas, XII = Other and unspecified malignant neoplasms

Coverage varied by diagnosis-based database, from 67.8% for CNS tumors to 76.9% for leukemia (Table 3), with some outliers depending on ICCC-3 diagnosis (Fig. 4a). The share of missing patients was higher in older age cohorts and earlier years of diagnosis (Fig. 4b and c).

**Table 3:** Coverage rate in the SALUB database

Selection criterion A			
Diagnosis databases	Number (n)	Number in SALUB (n)	Coverage (%)
Leukemia	1959	1507	76.9
Lymphoma	1244	869	69.9
CNS tumors	839	569	67.8
Solid tumors	1129	835	74.0
Total	5171	3780	73.1
Selection criterion B			
Leukemia	312	306	98.1
Lymphoma	188	172	91.5
CNS tumors	113	101	89.4
Solid tumors	240	224	93.3
Total	853	803	94.1

Coverage was calculated as the proportion of individuals meeting the respective selection criteria in each diagnosis-based database who were also registered in the SALUB database. Selection criteria A and B are defined in Table 1

The same coverage analysis was performed for patients born in 2002–2006, i.e. the subcohort expected to have been enrolled in the regular LTFU program that was in place in 2020, with an overall coverage rate of 94.1% (range 89,4% − 98,1%) (Table 3).

**Fig. 5:**
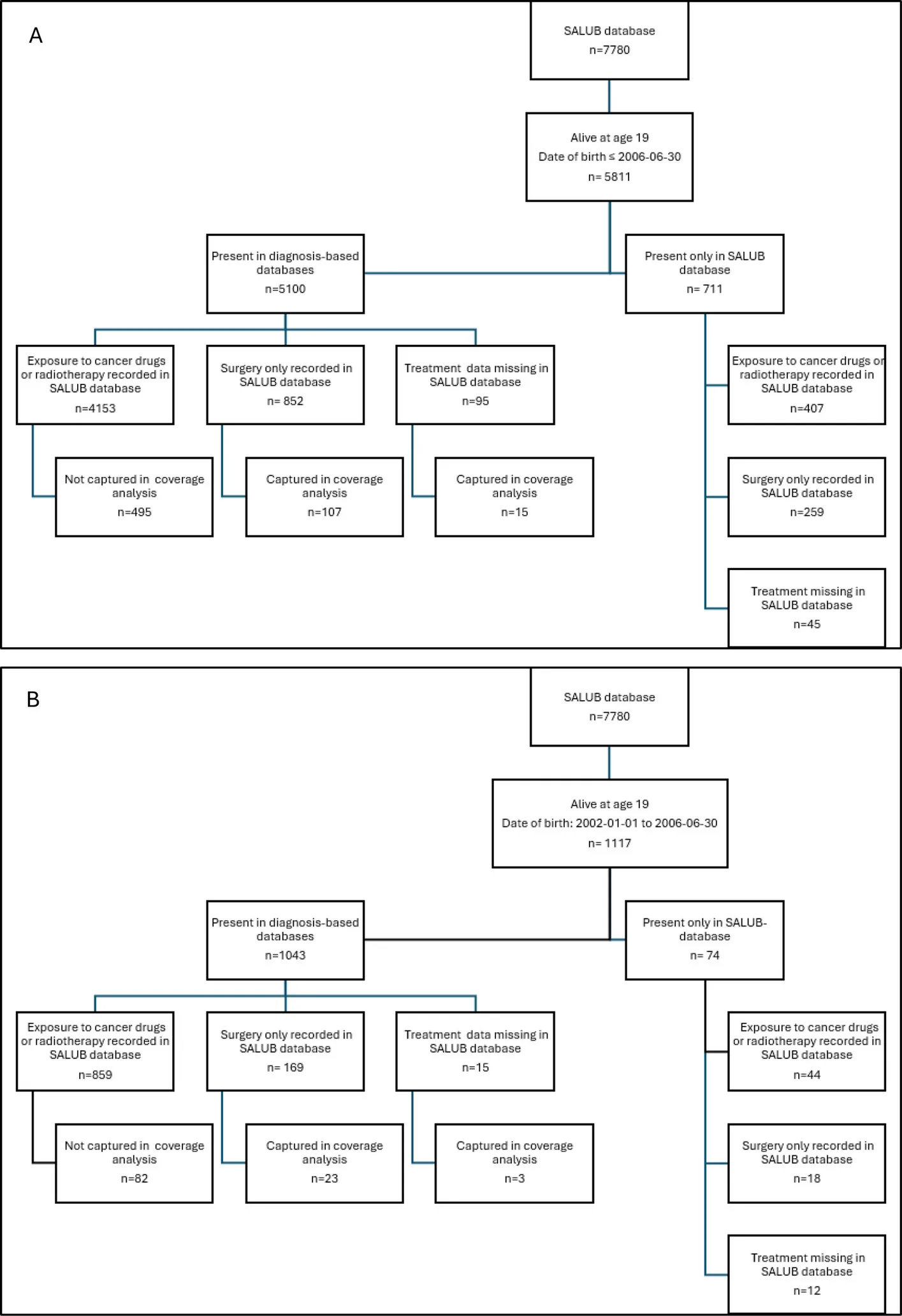
Assessment of the selection algorithm using data from the SALUB database. Flow diagrams showing individuals in the SALUB database who met the criteria for Selection A (**A**) and Selection B (**B**), stratified by presence in the diagnosis-based databases and treatment information recorded in the SALUB database

To evaluate the performance of the selection algorithm, the Selection A age criteria were applied directly to the SALUB database, identifying 5,811 individuals. Of these, 495 had systemic anticancer treatment and/or radiotherapy recorded in the SALUB database but had not been captured by the selection algorithm applied to the diagnosis-based databases. Conversely, 122 individuals captured by the selection algorithm had either surgery alone (*n* = 107) or no treatment information (*n* = 15) recorded in the SALUB database (Fig. 5). The corresponding figures when Selection B were applied revealed 82 individuals not captured in the diagnosis-based databases and 26 individuals erroneously captured. In a sensitivity analysis accounting for these discrepancies, estimated overall coverage changed from 73.1% (3,780/5,171) to 74.9% (4,153/5,544) for Selection A. For Selection B, estimated coverage changed from 94.1% (803/853) to 94.5% (859/909).

### Patients present exclusively in the SALUB-database (question 2)

Among individuals in the SALUB database meeting the Selection A age criteria, 711 were not present in the diagnosis-based modules. Among these, 407 had been treated with systemic anticancer treatment or radiotherapy and were therefore considered eligible for the LTFU program. Of these, 299 (73.6%) were born before 1993.

### Identification of certain risk groups (question 3)

#### Risk of gonadal toxicity

Using predefined exposure criteria (Table 2) in the SALUB database, we identified 582 females aged ≥ 19 years, who were considered at high risk of gonadal toxicity due to high cumulative doses of alkylating agents or radiotherapy with probable gonadal exposure (Table 4).

**Table 4:** Patients with high risk of gonadal toxicity and moderate and high risk of cardiotoxicity

High risk of gonadal toxicity		
Age groups (year of birth)	Female gender (n)	Male gender (n)
1953–1962	6	5
1963–1972	41	40
1973–1982	82	171
1983–1992	157	265
1993–2002	221	419
2003–2006*	75	160
Total	582	1060
Risk of cardiotoxicity		
Age groups (year of birth)	High risk (n)	Moderate risk (n)
1953–1962	8	-
1963–1972	68	25
1973–1982	224	216
1983–1992	330	447
1993–2002	522	613
2003–2006*	198	185
Sub total	1350	1486
Total	2836	

The number (n) of patients in the SALUB database meeting pre-defined treatment exposure criteria for high risk of gonadal toxicity or moderate-to-high risk of cardiotoxicity, as specified in Table 2. *: birth date no later than 30/06/2006

For males, sex-specific exposure criteria (Table 2) identified 1,060 individuals aged ≥ 19 years who were considered at high risk of impaired fertility, (Table 4).

#### Risk of cardiotoxicity

Applying predefined exposure criteria for risk of cardiotoxicity (Table 2), we identified 1,350 patients in the SALUB database who were classified as high risk and 1,486 who were classified as moderate risk (Table 4).

### Follow-up data

A total of 1,809 patients had at least one registered follow-up visit meeting the inclusion criteria. The completeness of variables describing diseases or conditions at follow-up varied markedly across organ systems, from 14% for urinary bladder disease to 100% for heart disease. The proportion of responses recorded as “unknown” also varied substantially, from 0.4% (bladder disease) to 19.5% (oral cavity conditions), (Supplementary material. Completeness of follow-up forms in the SALUB database in the Swedish childhood cancer quality registry).

Completeness of symptom variables also varied substantially across domains. The sexual function variable was completed in 99.9% of eligible follow-up records, while 63.3% of the responses were recorded as “unknown.” In contrast, the cardiac symptom variable was completed in 14.0% of records, with 2.6% recorded as “unknown” (Supplementary material. Completeness of follow-up forms in the SALUB database in the Swedish childhood cancer quality registry).

## Discussion

The SALUB database includes a substantial proportion of individuals treated with systemic anticancer therapy or radiotherapy for childhood cancer in Sweden, with particularly high coverage in younger cohorts. By integrating treatment exposure data with long-term follow-up outcome, the database provides a basis for generating individualized survivorship passports, risk-based follow-up, health care quality monitoring, and research.

The high coverage in the younger age group is consistent with successful implementation of the national LTFU structure, as treatment data is mainly entered into the database when preparing for a long-term follow-up visit. In older age groups, a higher proportion of the patients are missing in the SALUB database, reflecting the fact that the database was not in place until 2012. Furthermore, the clinical organization with LTFU-ACC was not fully implemented in all parts of the country until the late 2010 s and thus a substantial proportion in the older age group. i.e. date of birth before 01/01/2002, have not even been offered an LTFU visit and are therefore not registered in the database. It should be noted that the coverage analysis was limited by incomplete information in the diagnosis-based databases. Unfortunately, the applied additional ICCC-3-diagnoses criteria could not fully compensate for these limitations. Consequently, the coverage rates presented here, 73.1% for the entire cohort and 94.1% for the younger age group, should be interpreted with caution. However, the sensitivity analysis resulted in only small changes in these estimates, to 74.9% and 94.5%, respectively.

Additionally, the coverage analysis identifies opportunities to improve the completeness of the SALUB database. More complete registration of survivors eligible for risk-based follow-up could facilitate implementation of national care program recommendations across the survivor population. Improved coverage would also strengthen the value of the SALUB database for population-based research on late effects and for identifying individuals potentially eligible for future research and interventions.

The substantial number of patients registered in the SALUB database but not in the diagnosis-based databases of the Swedish childhood cancer quality registry was unexpected. This discrepancy may partly reflect the historical development of the registry, which was established in the early 1980 s and may have had lower coverage during its early years. Another possible explanation is the later development of adult LTFU services, through which long-term survivors may be entered in the SALUB database when requesting a treatment summary. Consistent with these explanations, most individuals with relevant treatment exposures who were registered exclusively in the SALUB database were born before 1993, although discrepancies were also observed in younger cohorts. These findings may help identify gaps in the diagnosis-based modules and inform future data-quality improvement.

The present study illustrates how treatment exposure data in the SALUB database can be used to identify groups of survivors meeting predefined risk criteria for specific late effects, exemplified here by cardiotoxicity and gonadal toxicity. As evidence and international follow-up guidelines evolve, updated risk definitions can be applied to the registered treatment data to estimate the number of survivors affected by changes in surveillance recommendations. When international guidelines for LTFU are updated, the data from the database could be used for calculations of possible needs for additional resource allocation measures, depending on the number of patients in a newly defined risk group. As an example, the updated 2023 IGHG guidelines on cardiomyopathy recommend life-long surveillance of the moderate risk group [11]. The national care program was revised accordingly and applying the new risk group stratification on SALUB data revealed a two to three-fold increase in the number of individuals recommended regular heart examinations. As follow-up data become more complete, the ability to identify and follow specific risk groups over time may also support evaluation of the implementation and outcomes of the national follow-up program. For researchers, SALUB can provide detailed treatment exposure data. as well as facilitate study planning by providing estimates of the number of potentially eligible participants.

The present study demonstrates that follow-up data in the SALUB database are not yet sufficiently complete or mature to allow reliable evaluation of the outcomes of the national care program surveillance recommendations. The data are also not yet sufficiently complete to assess adherence to organ-specific surveillance recommended in the national care program. The follow-up form has undergone several revisions since its introduction in 2022, with variables for additional organ systems added over time. Consequently, variables introduced more recently have lower completeness than those included since 2022. Completeness is expected to improve as more patients attend subsequent LTFU visits and the updated forms are completed. Differences in the proportion of responses recorded as “unknown” may also reflect variation in how routinely specific health domains are assessed and documented during LTFU visits. For example, oral health and sexual function may not be systematically addressed at every visit.

The workload required for complete and accurate registration is substantial and represents an important implementation challenge. Improving data completeness will require either dedicated resources for registry data entry or greater automation, including transfer of relevant data from electronic health records and integration of patient-reported outcome measures (PROMs).

This report highlights the challenges involved in developing a population-based registry containing detailed treatment and long-term follow-up data. By describing the current content, coverage, and limitations of the SALUB database, it provides a basis for future data-quality improvement efforts and facilitates appropriate interpretation of the registry data within research. Finally, the SALUB database is an integral part of long-term follow-up of Swedish childhood cancer survivors, enabling risk-based screening and follow-up care. From a health care perspective, a national registry may also support more equitable delivery of survivorship care by identifying gaps in coverage and enabling follow-up needs to be quantified across patient groups and regions. Such information can inform resource planning and support continuity during the transition from pediatric to adult care. However, achieving these goals depends on complete and timely registration across all centers, highlighting the need for sustainable resources and standardized registration practices.

## Conclusions

The SALUB database has high coverage among younger childhood cancer survivors and enables identification of individuals according to treatment-defined risks of late effects. However, lower coverage in older cohorts and incomplete follow-up data currently limit some applications, including evaluation of adherence to and outcomes of national long-term follow-up recommendations. Continued development and improved completeness of the database may strengthen its role in risk-based survivorship care, health care planning, and research.

## Supplementary Information

Supplementary Material 1.

## Data Availability

The datasets generated and/or analyzed during the current study are not publicly available due to the national privacy/data integrity regulations but are available from the corresponding author on reasonable request.

## Abbreviations

SALUB: Swedish working group for long-term follow-up after childhood cancer
SALUB database: The long-term follow-up section of the Swedish childhood cancer quality registry (the Swedish National Childhood Cancer Registry)
LTFU: Long-term follow-up
LTFU-ACC: Long-term follow-up clinic for adults after childhood cancer
IGHG: International Guideline Harmonization Group for Late Effects of Childhood Cancer
IQR: Inter quartile range
