# Supplementary material for: The Swedish childhood cancer quality registry for risk-based long-term follow-up after childhood cancer: structure, content, and development over time
Source: Arch Public Health. 2026 Nov 9;84:248. doi: 10.1186/s13690-026-02136-2 (PMC13760745; doi:10.1186/s13690-026-02136-2)
Supplement: Supplementary file 1 [file 13690_2026_2136_MOESM1_ESM.pdf]

Supplementary material

Completeness of follow-up forms in the SALUB database in the Swedish childhood cancer quality registry.

A. Completeness of registered diseases/disorders in follow-up forms in the SALUB database in the Swedish childhood cancer quality registry. Data extraction date 30/06/2025. *

Organ system or functional domains	Year of birth						
	1953–2006	1953–1962	1963–1972	1973–1982	1983–1992	1993–2002	2003–2006
Endocrine, n (%)	1709 (94.5)	6 (100.0)	68 (93.2)	215 (95.1)	356 (94.7)	646 (96.3)	418 (91.5)
Endocrine unknown, n (%)	92 (5.1)	1 (16.7)	1 (1.4)	9 (4.0)	27 (7.2)	43 (6.4)	11 (2.4)
Heart, n (%)	1809 (100.0)	6 (100.0)	73 (100.0)	226 (100.0)	376 (100.0)	671 (100.0)	457 (100.0)
Heart unknown, n (%)	117 (6.5)	0 (0.0)	4 (5.5)	12 (5.3)	39 (10.4)	47 (7.0)	15 (3.3)
Urinary bladder, n (%)	253 (14.0)	0 (0.0)	13 (17.8)	38 (16.8)	56 (14.9)	116 (17.3)	30 (6.6)
Urinary bladder unknown, n (%)	7 (0.4)	0 (0.0)	0 (0.0)	4 (1.8)	1 (0.3)	2 (0.3)	0 (0.0)
Neuropsychiatry, n (%)	422 (23.3)	0 (0.0)	15 (20.5)	46 (20.4)	85 (22.6)	184 (27.4)	92 (20.1)
Neuropsychiatry unknown, n (%)	26 (1.4)	0 (0.0)	0 (0.0)	4 (1.8)	10 (2.7)	9 (1.3)	3 (0.7)
Oral cavity, n (%)	1800 (99.5)	6 (100.0)	73 (100.0)	225 (99.6)	374 (99.5)	669 (99.7)	453 (99.1)
Oral cavity unknown, n (%)	352 (19.5)	2 (33.3)	13 (17.8)	69 (30.5)	92 (24.5)	141 (21.0)	35 (7.7)
Second malignancy, n (%)	1805 (99.8)	6 (100.0)	73 (100.0)	225 (99.6)	376 (100.0)	669 (99.7)	456 (99.8)
Second malignancy unknown, n (%)	95 (5.3)	0 (0.0)	2 (2.7)	14 (6.2)	22 (5.9)	42 (6.3)	15 (3.3)
Fertility, n (%)	254 (14.0)	0 (0.0)	13 (17.8)	38 (16.8)	57 (15.2)	116 (17.3)	30 (6.6)
Fertility unknown, n (%)	39 (2.2)	0 (0.0)	2 (2.7)	3 (1.3)	11 (2.9)	16 (2.4)	7 (1.5)
Sexual function, n (%)	550 (30.4)	1 (16.7)	25 (34.2)	84 (37.2)	117 (31.1)	213 (31.7)	110 (24.1)
Sexual function unknown, n (%)	73 (4.0)	0 (0.0)	2 (2.7)	10 (4.4)	21 (5.6)	35 (5.2)	5 (1.1)
Vascular, n (%)	548 (30.3)	1 (16.7)	25 (34.2)	84 (37.2)	116 (30.9)	212 (31.6)	110 (24.1)
Vascular unknown, n (%)	54 (3.0)	0 (0.0)	0 (0.0)	9 (4.0)	15 (4.0)	25 (3.7)	5 (1.1)
Gastrointestinal, n (%)	752 (41.6)	3 (50.0)	41 (56.2)	113 (50.0)	184 (48.9)	287 (42.8)	124 (27.1)
Gastrointestinal unknown, n (%)	161 (8.9)	0 (0.0)	5 (6.8)	29 (12.8)	43 (11.4)	64 (9.5)	20 (4.4)
Mental health, n (%)	1804 (99.7)	6 (100.0)	73 (100.0)	226 (100.0)	375 (99.7)	669 (99.7)	455 (99.6)
Mental health unknown, n (%)	210 (11.6)	0 (0.0)	7 (9.6)	32 (14.2)	63 (16.8)	78 (11.6)	30 (6.6)
Blood pressure, n (%)	253 (14.0)	0 (0.0)	13 (17.8)	38 (16.8)	56 (14.9)	116 (17.3)	30 (6.6)
Blood pressure unknown, n (%)	36 (2.0)	0 (0.0)	3 (4.1)	8 (3.5)	9 (2.4)	15 (2.2)	1 (0.2)

Lung, n (%)	667 (36.9)	4 (66.7)	35 (47.9)	102 (45.1)	158 (42.0)	246 (36.7)	122 (26.7)
Lung unknown, n (%)	103 (5.7)	2 (33.3)	1 (1.4)	16 (7.1)	29 (7.7)	38 (5.7)	17 (3.7)
Kidney, n (%)	692 (38.3)	1 (16.7)	36 (49.3)	101 (44.7)	151 (40.2)	256 (38.2)	147 (32.2)
Kidney unknown, n (%)	127 (7.0)	0 (0.0)	7 (9.6)	23 (10.2)	35 (9.3)	37 (5.5)	25 (5.5)
Liver, n (%)	564 (31.2)	1 (16.7)	28 (38.4)	81 (35.8)	127 (33.8)	223 (33.2)	104 (22.8)
Liver unknown, n (%)	144 (8.0)	1 (16.7)	5 (6.8)	23 (10.2)	39 (10.4)	58 (8.6)	18 (3.9)
Bile ducts, n (%)	564 (31.2)	1 (16.7)	28 (38.4)	81 (35.8)	127 (33.8)	223 (33.2)	104 (22.8)
Bile ducts unknown, n (%)	142 (7.8)	1 (16.7)	5 (6.8)	20 (8.8)	39 (10.4)	60 (8.9)	17 (3.7)
Pancreas, n (%)	564 (31.2)	1 (16.7)	28 (38.4)	81 (35.8)	127 (33.8)	223 (33.2)	104 (22.8)
Pancreas unknown, n (%)	144 (8.0)	1 (16.7)	4 (5.5)	23 (10.2)	40 (10.6)	59 (8.8)	17 (3.7)
Neurology, n (%)	790 (43.7)	3 (50.0)	43 (58.9)	118 (52.2)	185 (49.2)	296 (44.1)	145 (31.7)
Neurology unknown, n (%)	110 (6.1)	1 (16.7)	3 (4.1)	18 (8.0)	26 (6.9)	43 (6.4)	19 (4.2)
Ear/hearing, n (%)	977 (54.0)	3 (50.0)	46 (63.0)	143 (63.3)	238 (63.3)	364 (54.2)	183 (40.0)
Ear/hearing unknown, n (%)	209 (11.6)	1 (16.7)	8 (11.0)	31 (13.7)	64 (17.0)	84 (12.5)	21 (4.6)
Eye/vision, n (%)	981 (54.2)	2 (33.3)	45 (61.6)	148 (65.5)	224 (59.6)	374 (55.7)	188 (41.1)
Eye/vision unknown, n (%)	180 (10.0)	1 (16.7)	8 (11.0)	29 (12.8)	47 (12.5)	78 (11.6)	17 (3.7)
Bone and soft tissue, n (%)	1803 (99.7)	6 (100.0)	72 (98.6)	226 (100.0)	376 (100.0)	669 (99.7)	454 (99.3)
Bone and soft tissue unknown, n (%)	187 (10.3)	1 (16.7)	9 (12.3)	27 (11.9)	55 (14.6)	75 (11.2)	20 (4.4)
Breast, n (%)	252 (13.9)	0 (0.0)	13 (17.8)	38 (16.8)	56 (14.9)	116 (17.3)	29 (6.3)
Breast unknown, n (%)	3 (0.2)	0 (0.0)	0 (0.0)	1 (0.4)	2 (0.5)	0 (0.0)	0 (0.0)
Skin, n (%)	1804 (99.7)	6 (100.0)	73 (100.0)	226 (100.0)	375 (99.7)	670 (99.9)	454 (99.3)
Skin unknown, n (%)	209 (11.6)	1 (16.7)	7 (9.6)	29 (12.8)	59 (15.7)	93 (13.9)	20 (4.4)
Hematology, n (%)	1804 (99.7)	6 (100.0)	73 (100.0)	225 (99.6)	375 (99.7)	670 (99.9)	455 (99.6)
Hematology unknown, n (%)	162 (9.0)	1 (16.7)	5 (6.8)	21 (9.3)	43 (11.4)	75 (11.2)	17 (3.7)
Immunology, n (%)	1802 (99.6)	6 (100.0)	73 (100.0)	225 (99.6)	376 (100.0)	669 (99.7)	453 (99.1)
Immunology unknown, n (%)	166 (9.2)	0 (0.0)	5 (6.8)	25 (11.1)	44 (11.7)	76 (11.3)	16 (3.5)
Total number, n	1809	6	73	226	376	671	457

*The completeness in total per different birth cohorts concerning disease in organ systems, filled out as YES, NO or UNKNOWN, with the total percentage of the variable coded as unknown below each organ system. Data is presented as number and percentage. Data is recorded routinely in follow-up forms based on information in patient records at regular medical visits at the long-term follow-up clinic.

B. Completeness of registered symptoms in follow-up forms in the SALUB database in the Swedish childhood cancer quality registry. Data extraction date 30/06/2025. *

Organ or functional domains	Birth-year intervals						
	1953–2006	1953–1962	1963–1972	1973–1982	1983–1992	1993–2002	2003–2006
Sexual function, n (%)	1807 (99.9)	6 (100.0)	73 (100.0)	226 (100.0)	376 (100.0)	670 (99.9)	456 (99.8)
Sexual function unknown, n (%)	1145 (63.3)	5 (83.3)	46 (63.0)	136 (60.2)	237 (63.0)	438 (65.3)	283 (61.9)
Breast, n (%)	548 (30.3)	1 (16.7)	25 (34.2)	84 (37.2)	116 (30.9)	213 (31.7)	109 (23.9)
Breast unknown, n (%)	92 (5.1)	0 (0.0)	2 (2.7)	15 (6.6)	26 (6.9)	39 (5.8)	10 (2.2)
Gastrointestinal, n (%)	753 (41.6)	3 (50.0)	41 (56.2)	114 (50.4)	184 (48.9)	287 (42.8)	124 (27.1)
Gastrointestinal unknown, n (%)	163 (9.0)	0 (0.0)	3 (4.1)	33 (14.6)	43 (11.4)	52 (7.7)	32 (7.0)
Mental health, n (%)	1710 (94.5)	6 (100.0)	68 (93.2)	215 (95.1)	357 (94.9)	645 (96.1)	419 (91.7)
Mental health unknown, n (%)	250 (13.8)	0 (0.0)	6 (8.2)	47 (20.8)	64 (17.0)	91 (13.6)	42 (9.2)
Neuropsychology, cognition, n (%)	1436 (79.4)	4 (66.7)	55 (75.3)	184 (81.4)	313 (83.2)	552 (82.3)	328 (71.8)
Neuropsychology, cognition unknown, n (%)	196 (10.8)	1 (16.7)	4 (5.5)	27 (11.9)	45 (12.0)	71 (10.6)	48 (10.5)
Heart, n (%)	254 (14.0)	0 (0.0)	13 (17.8)	38 (16.8)	57 (15.2)	116 (17.3)	30 (6.6)
Heart unknown, n (%)	47 (2.6)	0 (0.0)	2 (2.7)	11 (4.9)	11 (2.9)	21 (3.1)	2 (0.4)
Lung, n (%)	667 (36.9)	4 (66.7)	34 (46.6)	102 (45.1)	158 (42.0)	247 (36.8)	122 (26.7)
Lung unknown, n (%)	162 (9.0)	2 (33.3)	8 (11.0)	24 (10.6)	45 (12.0)	56 (8.3)	27 (5.9)
Neurology, n (%)	790 (43.7)	3 (50.0)	43 (58.9)	118 (52.2)	185 (49.2)	296 (44.1)	145 (31.7)
Neurology unknown, n (%)	151 (8.3)	2 (33.3)	5 (6.8)	31 (13.7)	36 (9.6)	49 (7.3)	28 (6.1)
Ear and hearing, n (%)	682 (37.7)	3 (50.0)	32 (43.8)	104 (46.0)	160 (42.6)	273 (40.7)	110 (24.1)
Ear and hearing unknown, n (%)	501 (27.7)	2 (33.3)	23 (31.5)	77 (34.1)	121 (32.2)	203 (30.3)	75 (16.4)
Eye and vision, n (%)	980 (54.2)	2 (33.3)	45 (61.6)	148 (65.5)	224 (59.6)	373 (55.6)	188 (41.1)
Eye and vision unknown, n (%)	556 (30.7)	2 (33.3)	22 (30.1)	90 (39.8)	138 (36.7)	205 (30.6)	99 (21.7)
Bone and soft tissue, n (%)	1806 (99.8)	6 (100.0)	73 (100.0)	226 (100.0)	376 (100.0)	669 (99.7)	456 (99.8)
Bone and soft tissue unknown, n (%)	172 (9.5)	2 (33.3)	10 (13.7)	28 (12.4)	39 (10.4)	65 (9.7)	28 (6.1)
Oral cavity, n (%)	1806 (99.8)	6 (100.0)	73 (100.0)	226 (100.0)	375 (99.7)	670 (99.9)	456 (99.8)
Oral cavity unknown, n (%)	705 (39.0)	3 (50.0)	35 (47.9)	125 (55.3)	175 (46.5)	238 (35.5)	129 (28.2)
Skin, n (%)	1807 (99.9)	6 (100.0)	73 (100.0)	226 (100.0)	376 (100.0)	670 (99.9)	456 (99.8)

Skin unknown, n (%)	499 (27.6)	2 (33.3)	24 (32.9)	74 (32.7)	119 (31.6)	192 (28.6)	88 (19.3)
Urinary bladder, n (%)	587 (32.4)	2 (33.3)	27 (37.0)	95 (42.0)	143 (38.0)	221 (32.9)	99 (21.7)
Urinary bladder unknown, n (%)	162 (9.0)	0 (0.0)	5 (6.8)	38 (16.8)	42 (11.2)	50 (7.5)	27 (5.9)
Total number, n	1809	6	73	226	376	671	457

The completeness in total per different birth cohorts concerning symptoms in organ systems, filled out as YES, NO or UNKNOWN, with the total percentage of the variable coded as unknown below each organ system. Data is presented as number and percentage (%). Data is recorded routinely in follow-up forms based on information in patient records at regular medical visits at the long-term follow-up clinic.
